# Partitioning of Selected Anisole and Veratrole Derivatives between Water and Anionic Surfactant Micelles

**DOI:** 10.3390/molecules25245818

**Published:** 2020-12-09

**Authors:** Andrzej Lewandowski, Katarzyna Szymczyk

**Affiliations:** Department of Interfacial Phenomena, Institute of Chemical Sciences, Faculty of Chemistry, Maria Curie-Skłodowska University in Lublin, Maria Curie-Skłodowska Sq. 3, 20-031 Lublin, Poland; andrzej.lewandowski@poczta.umcs.lublin.pl

**Keywords:** anisole and veratrole derivatives, SLS, SLES, micelle-water partitioning, APN model

## Abstract

The UV absorption spectra of six structurally related derivatives of anisole and veratrole, i.e., anisaldehyde, (E)-anethole, estragole, veratraldehyde, methyleugenol and (E)-methylisoeugenol, were recorded at various concentrations of the anionic surfactants, either sodium lauryl sulfate (SLS) or sodium lauryl ether sulfate (SLES) at T = 298 K. In addition, conductivity and density measurements were made for the SLS and SLES solutions to determine the volumetric properties of the studied surfactants. Next, using the W. Al-Soufi, L. Pińeiro and M. Novo model (APN model) including the pseudo-phase model for micellar solubilization, the values of micelle-water partition coefficients for each perfume-surfactant system were determined. In addition, the relations between the molecular structures of the solute and the head group of the surfactant and the value of the micelle-water partition coefficient as well as the octanol-water one were discussed.

## 1. Introduction

The efficiency of perfume raw materials (PRMs) in consumer products is greatly affected by their interactions with other components of the final product. This is related to both the chemical stability of PRMs in a final product matrix and their ability to evaporate from the matrix during the final product use [1,2]. In the case of liquid detergents, this matrix can be considered as a semidilute aqueous solution of surfactants, usually combined with a variety of additional ingredients such as viscosity modifiers, pigments, plant extracts, sequestrants or antibacterial ingredients [3,4,5,6]. Typical liquid detergent formulations, except those having extreme pH values or containing highly active components, such as oxidizing agents, do not usually cause serious problems related to the chemical stability of most commonly used PRMs. On the other hand, the ability of different PRMs to evaporate from a typical liquid detergent matrix varies considerably. This is mainly related to the differences in their vapor pressures and the extent of their interactions with the surfactants present in the matrix. From this point of view, understanding how the perfume interacts with surfactants in their aqueous solution is essential for many industrial applications of perfumes. It is known that in the aqueous solutions at concentrations which are higher than their critical micelle concentration (CMC), surfactants form micelles [7]. Since the solubilization of lipophilic solutes in the surfactant micelles leads to a decrease in their thermodynamic activity, the efficiency of perfumes in surfactant-based products is significantly affected by the partitioning of perfume raw materials between the surfactant micelles and the bulk aqueous phase [8,9,10,11]. The distribution of lipophilic solutes in the aqueous solutions of surfactants can be adequately described by the value of the micelle-water partition coefficient, $K_{MW}$, defined as the ratio of the concentration of a solute in a micellar pseudophase to its concentration in the bulk aqueous phase. The value of $K_{MW}$ depends on many factors, but mostly on the lipophilicity and the molecular structure of the solute as well as the molecular structure of the surfactant [9,11].

**Figure 1:**
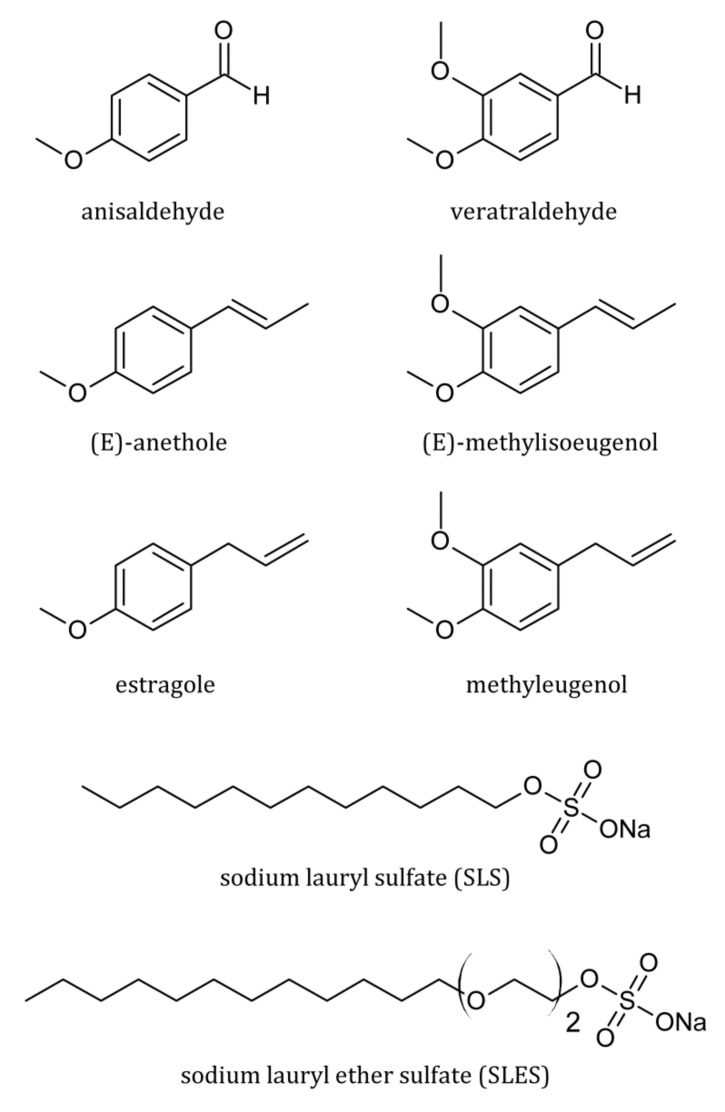
The molecular structure of studied fragrance materials and surfactants.

Modern liquid detergent formulations comprise a mixture of surfactants, mainly anionic, amphoteric and nonionic [12]. Among anionic ones, sodium lauryl sulfate (SLS) and sodium lauryl ether sulfate (SLES) are the lowest-cost and most widely used surfactants in consumer products for body care, such as shower gels, liquid hand soaps or shampoos, even though their safety has been the focus of numerous scientific studies [13,14,15,16,17]. On the other hand, scientific reports on the micellar partitioning of perfume raw materials in the aqueous SLES solution in comparison to its nonoxyethylenated counterpart (SLS) are scarce [11]. Taking this into account, this paper aimed at investigating the effect of lipophilicity of a solute and molecular structure of the head group of the anionic surfactants, Texapon^®^ LS 30 (SLS) and Texapon^®^ N 70 (SLES), on micelle-water partitioning of selected derivatives of anisole and veratrole (Figure 1), which are widely used in perfumery. For this purpose, UV spectra were recorded for each pair of perfume-surfactant systems at T = 298 K. Next, the W. Al-Soufi, L. Pińeiro and M. Novo model (APN model) [18] was used to determine the micelle-water partition coefficient ($K_{MW}$). In addition, conductivity and density measurements were made for the solutions of anionic surfactants to determine their volumetric properties needed for the determination of $K_{MW}$.

## 2. Results

According to the W. Al-Soufi, L. Pińeiro and M. Novo (APN) model [18,19], the concentration of monomeric surfactant, $C_{S,1}$, is approximately equal to the total surfactant concentration, $C_{S,0}$, below the CMC. Above the CMC, the concentration of monomeric surfactant is constant and equal to the CMC value. Close to the CMC value, the concentration of surfactant monomers changes more or less gradually from the $C_{S,0}$ to the CMC value. On the other hand, the concentration of aggregated (micellized) surfactants, $C_{S,M}$, can be calculated as the difference between the total concentration and that of monomeric surfactants in the whole range of total surfactant concentration:

(1) $$C_{S,M}=C_{S,0}-C_{S,1}$$

Assuming that the second derivative of the concentration of monomeric surfactant, $C_{S,1}$, with respect to the total surfactant concentration can be described by the Gauss function centered at the CMC and with the width σ, Al-Soufi, Pińeiro and Novo derived the following equation for the variation of $C_{S,1}$ with $C_{S,0}$ (see Ref. [18] for a detailed derivation):

(2) $$C_{S,1}=CMC\left[1-\frac{A}{2}\left(\sqrt{\frac{2}{\pi }r\cdot e^{\frac{{\left(\frac{C_{S,0}}{CMC}-1\right)}^{2}}{2r^{2}}}}+\left(\frac{C_{S,0}}{CMC}-1\right)\left(erf\left(\frac{\frac{C_{S,0}}{CMC}-1}{\sqrt{2}\cdot r}\right)-1\right)\right)\right]$$

where

(3) $$A=\frac{2}{1+\sqrt{\frac{2}{\pi }}r\cdot e^{\frac{-1}{2r^{2}}}+erf\left(\frac{1}{\sqrt{2}\cdot r}\right)}$$

and r=σ/CMC is called the relative micellar transition width and is a measure of the width of the concentration range in the transition region around the CMC [18].

**Figure 2:**
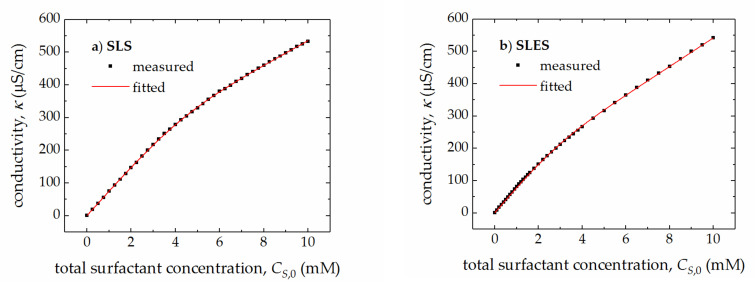
The measured and fitted values of electrical conductivity, κ, of the aqueous solutions of SLS (**a**) and SLES (**b**) vs. the total surfactant concentration, $C_{S,0}$.

Taking into account the measured values of conductivity, κ, of the aqueous solutions of studied surfactants (Figure 2a,b), the parameters of the APN model (CMC and *r*) were determined (Table 1) by fitting the κ values to the Equations (1)–(3) for the monomer and micellar concentrations in the surfactant solution assuming additivity of conductivities of the monomeric and micellized surfactant, that is:

(4) $$\kappa =\kappa_{W}+a\cdot C_{S,1}+b\cdot C_{S,M}$$

where $\kappa_{W}$ is the residual conductivity of the solvent (originating, for instance, from the dissolution of CO_2_ and autoionization of water). The specific conductivity of water used in sample preparation was determined as 0.78 µS/cm. The empirical parameters a and b represent the limiting slopes of the conductivity-concentration curve below and above the CMC, respectively. The values of parameters a and b determined from the fits are given in Table 1.

**Figure 3:**
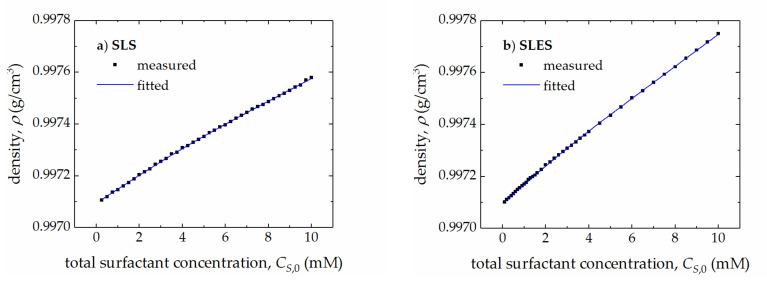
The measured and fitted values of density, ρ, of the aqueous solutions of SLS (**a**) and SLES (**b**) vs. the total surfactant concentration, $C_{S,0}$.

Next, taking into account the measured values of density, ρ, of the aqueous solutions of SLS and SLES (Figure 3a,b) and assuming that the volumetric properties of the solvent remain constant in the limited concentration range of the surfactant, the values of apparent molar volumes of momoneric ($V_{S,1}^{\phi }$) and micellized ($V_{S,M}^{\phi }$) surfactants were determined (Table 1) by using Equations (1)–(3) and the following equation:

(5) $$\rho =\rho_{W}+\left(M_{S}-\rho_{W}\cdot V_{S,1}^{\phi }\right)\cdot C_{S,1}+\left(M_{S}-\rho_{W}\cdot V_{S,M}^{\phi }\right)\cdot C_{S,M}$$

where $\rho_{W}$ is the density of water (0.99707 g/cm^3^ at 298 K) and $M_{S}$ is the molar mass of surfactant.

As results from Table 1 show, the obtained values of CMC of surfactants are somewhat smaller than those presented in the literature, but those of apparent molar volumes are almost the same [20,21,22,23,24,25,26]. The CMC of SLES is lower than that of SLS, which is related to the incorporation of the ethylene oxide (EO) group between the lauryl hydrophobic group and the hydrophilic moiety and as a result of reduction of the degree of EO group’s hydration due to the presence of the sulfate group. In addition, the attractive ion-dipole interaction between SO_4_^−^ and nearby O → CH_2_ dipole of the EO group results in a significant reduction in the electrostatic repulsion of the head groups and lowers SLES CMC [27,28].

The interactions between surfactants and perfume materials have been studied from various aspects and by many analytical methods [11,12,29,30,31]. In our studies, the values of the micelle-water partition coefficient, $K_{MW}$, for each pair of the perfume-surfactant system were determined by fitting the UV spectra of the studied fragrance materials obtained at all concentrations of surfactant to the equations of the APN model (Equations (1)–(3)) and the relation between the absorbance (Abs) of the solution and the concentration of the micellized surfactant:

(6) $$Abs=Abs_{W}+\left(Abs_{M}-Abs_{W}\right)\cdot \frac{K_{MW}\cdot C_{S,M}\cdot V_{S,M}^{\phi }}{1+\left(K_{MW}-1\right)\cdot C_{S,M\cdot }V_{S,M}^{\phi }}$$

**Figure 4:**
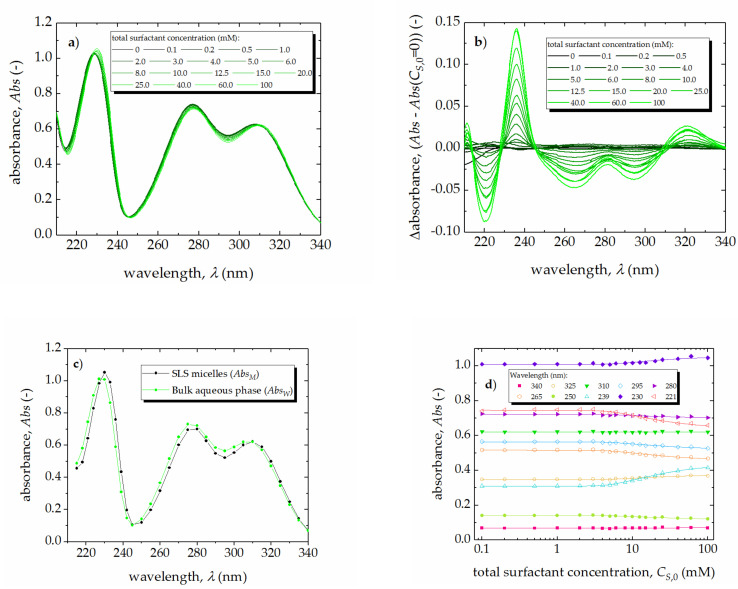
The effect of SLS concentration on the UV absorption spectrum of veratraldehyde: (**a**) experimental UV spectra; (**b**) differential UV spectra; (**c**) variation of absorbance at selected wavelengths; (**d**) fitted UV spectra.

The obtained UV spectra for all studied perfume-surfactant mixtures were fitted simultaneously at 30 selected wavelengths. An example of the results of fitting the model equations to the experimental data is given in Figure 4 for SLS and veratraldehyde. The results of calculations for each perfume-surfactant system are summarized in Table 2.

**Figure 5:**
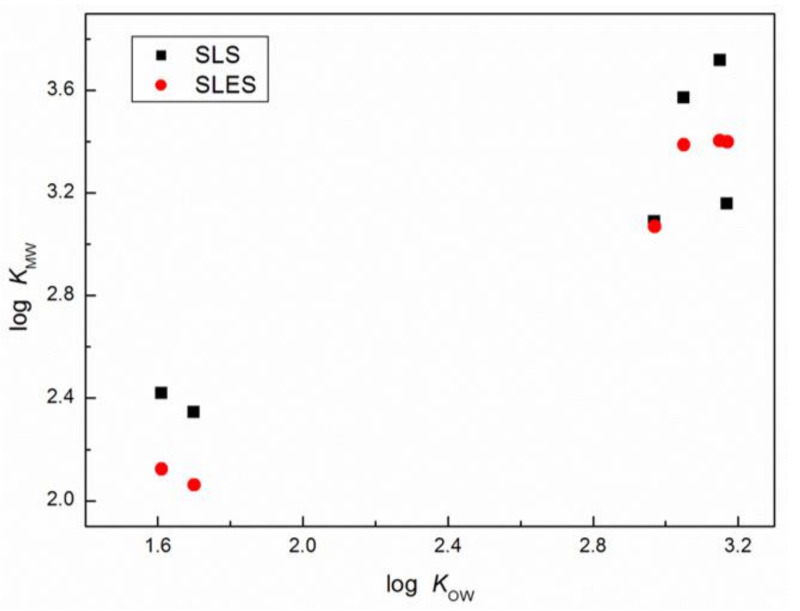
The values of log $\log K_{MW}$ vs. $\log K_{OW}$ for the studied fragrance materials and anionic surfactants.

The values of partition coefficients between the aqueous phase and SLS micelles were determined earlier for anisaldehyde and (E)-anethole by micellar electrokinetic chromatography. The $\log K_{MW}$values were reported to be equal to 2.3 and 3.22 [34,35] for anisaldehyde and (E)-anethole, respectively. Thus, the values of $\log K_{MW}$ reported here for these two perfume raw materials in the aqueous SLS solution are consistent with those reported earlier in the literature. As results from Table 2 show, all studied perfume molecules are incorporated into the surfactant micelle. In addition, for all perfumes except (E)-anethole, the values of $\log K_{MW}$ for SLS are higher than those for SLES, which suggests that for these perfumes, the solubilization site in the SLS micelles is energetically more favorable than in the case of the SLES micelles. This reduced molecule affinity for the SLES micelles may result from the more difficult packing of the surfactant and perfume molecules in the micelles of ethoxylated surfactant due to the incorporation of the bulky EO unit in the surfactant head group, which hinders the accommodation of guest molecules. These EO moieties together with those of –OSO_3_^−^ (surfactant polar head groups) can also separate to greater distances, opening up the micelle structure from a somewhat compact structure to a loose, disordered one [36,37]. On the other hand, if the oxyethylene units are restricted not only to the polar phase, as can be found in the literature, some of them are extended into the hydrocarbon phase of the micelle and can penetrate its hydrophobic core [38,39]. In addition, the oxyethylene group can be associated with two molecules of water, but different water species of varying strength and coordination participate in the hydration of the EO groups [37,40]. Maybe for this reason and the presence of anethole/water clusters, despite its low solubility in water [41], the values of $\log K_{MW}$ for (E)-anethole and SLES are higher than those for SLS (Table 2). Both for SLS and SLES, the biggest values of log$K_{MW}$ are found for estragole, but for the (E)-anethole-estragole isomer pair, which only differs by the location of the double bond in the propenyl chain, the values of $K_{MW}$ for SLES are the same (Table 2). In addition, the biggest values of log$K_{MW}$ are in accordance with the smallest value of topological polar surface area (9.2 Å^2^ [32]) and suggest that estragole and (E)-anethole molecules are located near the hydrophobic cores of SLS and SLES micelles. The values of $K_{MW}$ for the other solutes confirmed that the perfume may penetrate into the palisade layer of the studied surfactant micelles. From this point of view, it was interesting to compare the obtained values of $K_{MW}$ with those of the octanol-water partition coefficient, $K_{OW}$, by far the best-documented and most frequently used hydrophobicity parameter in different studies [42,43,44]. The values of $K_{OW}$ for the studied mixtures were calculated with ACD/ChemSketch 2012 (ACD Labs, Toronto, ON, Canada) and are presented in Table 2 together with those taken from the literature [32,33]. As results show from this table, all $K_{OW}$ values are smaller than the appropriate values of the micelle–water partition coefficient, and the biggest differences between $K_{MW}$ and $K_{OW}$ are observed for veratraldehyde. This indicates that veratraldehyde molecules may be located in the head group regions of SLS and SLES micelles. This is in accordance with the values of the topological polar surface area, which for veratraldehyde is the largest of all the fragrances tested and equal to 35.5 Å^2^ [32]. In the case of the isomer (E)-anethole-estragole pair, we can obviously notice that all presented values in Table 2 are almost the same except $\log K_{MW}$ for SLS, which is probably connected with the structure of mentioned fragrances. It should be remembered that restriction of the rotation of the double bond when located in the middle of the propenyl chain of (E)-anethole results in a planar, linear and rigid structure of this perfume, as opposed to estragole in which the double bond can undergo free rotation around the carbon-carbon single bond increasing the steric hindrance. Comparing the values in Table 2, it becomes obvious that the value of $K_{MW}$ for each studied surfactant increases with an increase of solute lipophilicity, but as results from Figure 5 show, this relation is not strictly linear. It means that in the studied systems the values of the micelle-water partition coefficient are influenced by both perfume hydrophobicity and specific solute-surfactant interactions as well as those between the solute and water molecules penetrating the palisade layer of the surfactant micelle.

## 3. Materials and Methods

Anisaldehyde (≥99%), (E)-anethole (≥99%), estragole (≥98%), veratraldehyde (≥99%), methyleugenol (≥99%) and (E)-methylisoeugenol (≥95%) were provided by Pollena-Aroma (Nowy Dwór Mazowiecki, Poland). Technical-grade surfactants: Texapon^®^ LS 30 (SLS, average $M_{S}$ = 297 g/mol) and Texapon^®^ N 70 (SLES, average $M_{S}$ = 382 g/mol) were kindly provided by BASF (Ludwigshafen, Germany) (Figure 1). All materials were used without further purification. UV absorption spectra were recorded with the U-2900 spectrophotometer (Hitachi, Tokyo, Japan) for the aqueous solutions of studied fragrance materials (5 × 10^−5^ mol/dm^3^) in the presence of studied surfactants (either SLS or SLES). The concentration of surfactants varied from 10^−4^ to 10^−1^ mol/dm^3^. Additionally, UV absorption spectra were recorded for the studied surfactant solutions without the addition of fragrance materials (background spectra). In order to evaluate the effect of surfactant concentration on the UV absorption spectra of studied fragrance materials, the background spectra were subtracted from those recorded for the surfactant-fragrance mixtures. Volumetric and electrical properties of studied surfactants were investigated by means of conductivity (S230 SevenCompact conductivity meter equipped with InLab 731 ISM probe, Mettler Toledo, Switzerland) and density (DMA 5000 density meter, Anton Paar, Austria) measurements of their aqueous solutions. All measurements were made at 298 K.

## 4. Conclusions

Using the UV absorption spectra of six structurally related derivatives of anisole and veratrole in the presence of anionic surfactants, SLS and SLES, as well as the measured values of density and conductivity of their aqueous solutions, the values of micelle-water partition coefficients for each perfume-surfactant system were determined. From the obtained data and calculations, it is evident that the value of the micelle-water partition coefficient increases with an increase of solute lipophilicity as determined by the $\log K_{OW}$ value. In addition, in most cases, higher values of $K_{MW}$ are observed for the SLS micelles than for the SLES micelles, which suggests that the solute solubilization site in the SLS micelles is energetically more favorable than in the case of SLES micelles. On the other hand, in the studied systems, the relation between the values of the micelle-water and octanol-water partition coefficient is not strictly linear, which suggests that the values of $K_{MW}$ are influenced not only by the hydrophobicity of the solute but also by the specific solute-surfactant and solute-water interactions.

## Figures and Tables

**Table 1:** The values of the W. Al-Soufi, L. Pińeiro and M. Novo (APN) model parameters and apparent molar volumes of sodium lauryl sulfate (SLS) and sodium lauryl ether sulfate (SLES) (all given uncertainties correspond to the standard errors of parameters obtained from the fits).

Surfactant	CMC (mM)	*r* (–)	a (S cm^2^/mol)	b (S cm^2^/mol)	$V_{S,1}^{\phi }$ (cm^3^/mol)	$V_{S,M}^{\phi }$ (cm^3^/mol)
SLS	4.17 ± 0.06	0.45 ± 0.02	75.6 ± 0.7	37.6 ± 0.4	243.5 ± 0.2	254.1 ± 0.2
SLES	2.74 ± 0.02	0.56 ± 0.03	80.1 ± 0.5	44.2 ± 0.3	308.2 ± 0.3	321.2 ± 0.2

**Table 2:** The logarithmic values of the calculated values of micelle-water, $K_{MW}$, and octanol-water, $K_{OW}$, partition coefficients for the studied fragrance materials as well as those taken from the literature, $K_{OW}$* [32,33] (the uncertainties of $K_{MW}$ values correspond to the standard errors of parameters obtained from the fits, while those of $K_{OW}$ were calculated with ACD/ChemSketch 2012 (ACD Labs, Toronto, ON, Canada)).

Materials	$\log K_{MW}$ (SLS)	$\log K_{MW}$ (SLES)	$\log K_{OW}$	$\log K_{OW}*$
Veratraldehyde	2.42 ± 0.02	2.12 ± 0.02	1.61 ± 0.27	1.22
Methyleugenol	3.09 ± 0.06	3.07 ± 0.06	2.97 ± 0.24	3.03
(E)-methylisoeugenol	3.57 ± 0.07	3.39 ± 0.07	3.05 ± 0.24	3.47
Anisaldehyde	2.34 ± 0.02	2.06 ± 0.02	1.70 ± 0.26	1.76
Estragole	3.72 ± 0.05	3.40 ± 0.05	3.15 ± 0.22	3.47
(E)-anethole	3.16 ± 0.05	3.40 ± 0.04	3.17 ± 0.22	3.40
